# Stratégie de prise en charge de l'hypertension artérielle en hémodialyse chronique: un modèle appliqué d’éducation thérapeutique des patients (ETP)

**DOI:** 10.11604/pamj.2014.19.86.3996

**Published:** 2014-09-25

**Authors:** Ryme El Harraqui, Abda Naima, Bentata Yassamine, Intissar Haddiya

**Affiliations:** 1 Service de Néphrologie-Hémodialyse, Centre Hospitalier Al Farabi, Oujda, Maroc; 2 Laboratoire de Biostatistiques, Faculté de Médecine et de Pharmacie, Université Mohamed I^er^, Oujda, Maroc

**Keywords:** Hypertension artérielle, éducation thérapeutique des patients, poids sec, prise de poids interdialytique, rythme de dialyse, régime hyposodé, règles hygiéno-diététique, modèle constructiviste, pédagogie allostérique, hypertension, patient education, dry weight sec, interdialytic weight gain, dialysis rate, low salt diet, lifestyle, constructivist model, allosteric learning model

## Abstract

Même après l'entrée en dialyse, la prévalence de l'hypertension artérielle (HTA) reste élevée. Elle est souvent greffée d'une forte morbi-mortalité, et altère la qualité de vie des patients hémodialysés chroniques. La « méthode du poids sec (PS) », établie par Scribner, présente un intérêt indéniable dans la gestion de l'HTA chez le patient hémodialysé. Le but de notre travail était de déterminer la prévalence et les facteurs de risque (FDR) de l'HTA chez nos hémodialysés chroniques et d'en tenter une réduction en nous basant sur une stratégie d’éducation thérapeutique basée sur les recommandations de Scribner. Nous avons mené une étude prospective interventionnelle en trois mois et en trois phases, auprès des 93 hémodialysés chroniques de l'Hôpital Al Farabi d'Oujda. En phase 1, nous avons déterminé la prévalence de l'HTA par la surveillance horaire de la tension artérielle (TA) durant la séance de dialyse pendant deux semaines, soit sur un total de 442 séances. Les patients ont également mesuré leur pression artérielle (PA) hors-centre de façon biquotidienne les jours de non-dialyse, soit un total de 1720 mesures hors-centre. L'HTA a été définie par une Pression Artérielle Systolique (PAS) supérieure ou égale à 140mmHg et/ou une Pression Artérielle Diastolique (PAD) supérieure ou égale à 90mmHg sur au moins 2 mesures. En phase 2, les patients hypertendus ont bénéficié d'une prise en charge basée sur les recommandations de Scribner et une éducation thérapeutique (ETP). En phase 3, nous évalué les retombées de notre prise en charge. Une HTA a été notée sur 231 séances chez 57 patients, soit une prévalence de 61,3%. La PAS moyenne était de 172,75±17,69 (145-220) mmHg. Les FDR retenus sont: l’âge, la PPID importante, le non respect des règles hygiéno-diététiques (RHD) et le rythme de 2 séances de dialyse/semaine. Au départ, 13 patients (22,8%) étaient dialysés trois fois par semaine; nous avons doublé ce taux. Nous avons également réduit le PS chez 48 patients, à raison de ½Kg tous les 15 jours. Les résultats en phase 3 montrent que 8 patients sont passés dans le groupe des patients «normotendus », la prévalence de l'HTA passant ainsi à 52,7%. Dans le nouveau groupe «HTA + » (n = 49), la PAS moyenne est passée à 166,12±17,05 (140-200) mmHg. La PPID a baissé de 0,98±0,425 (0,35-2,1) Kgs chez 46 patients. La prévalence de l'HTA dans notre population rejoint les données de la littérature, mais en appliquant les principes de l'ETP à la prise en charge de celle-ci, nous avons pu en baisser la prévalence. Ainsi, La « méthode du PS » peut permettre de corriger l'HTA en HD, mais ce succès ne doit se concevoir sans un effort pédagogique soutenu de la part de l'ensemble de l’équipe soignante, d'où l'intérêt de planifier une ETP

## Introduction

Il est actuellement bien établi que la prévention de l'hypertension artérielle (HTA) et de ses complications à court et long terme est l'un des buts essentiels de l'hémodialyse de suppléance (HD). Cependant, on constate que même après l'entrée en dialyse, la prévalence de celle-ci reste très élevée et greffée d'une forte morbi-mortalité. Scribner a établi très tôt une liste de recommandations dans le traitement non pharmacologique de l'HTA de l'hémodialysé chronique et que l'on regroupe désormais sous le terme de « méthode du poids sec ». Notre étude s'inscrit dans une volonté d'améliorer la prise en charge tensionnelle de nos patients hémodialysés chroniques, et cela à l'aide de moyens non pharmacologiques. Nous l'avons donc menée en trois étapes aux objectifs distincts: Ainsi, il s'agissait dans un premier temps de déterminer la prévalence de l'HTA et ses facteurs de risque (FDR) au sein de notre population d'hémodialysés chroniques. Puis, dans un second temps, nous avons tenté de réduire les chiffres tensionnels de nos patients en adoptant une stratégie d’éducation thérapeutique basée sur les recommandations de Scribner. Enfin, nous avons évalué les retombées de notre prise en charge non pharmacologique et de notre implication pédagogique.

## Méthodes

Notre travail a consisté en une étude prospective interventionnelle menée en trois mois, d'Octobre à Décembre 2011, et en trois phases, auprès des 93 hémodialysés chroniques du centre d'hémodialyse de l'Hôpital Al Farabi d'Oujda. La première phase a duré deux semaines et a consisté en l’établissement d'un état des lieux, un constat de la prévalence de l'HTA dans notre population basé sur deux volets: - d'une part, une surveillance horaire de la pression artérielle durant les séances d'hémodialyse, menée par l'ensemble du personnel médical et paramédical, à l'aide de tensiomètres manuels à brassards adaptés; - d'autre part, nous avons recommandé à nos patients d'effectuer, de façon biquotidienne, une mesure ambulatoire au repos de leur pression artérielle les jours de non-dialyse. En centre, nous avons surveillé la PA de tous nos patients au branchement, à chaque heure puis au débranchement (avant et 15 minutes après restitution) pendant une durée de deux semaines, ce qui revient à un total de 420 séances de dialyse sous étroite surveillance. Le personnel chargé des mesures de pression artérielle était constitué de six médecins et dix infirmiers répartis équitablement entre les deux salles de dialyse comportant chacune treize générateurs.

1720 mesures de pression artérielle hors-centres ont été colligées en deux semaines (10 mesures par semaine pour les patients dialysés deux fois et 8 pour ceux dialysés trois fois par semaine). Elles ont été effectuées, à domicile, en position allongée, par les patients eux-mêmes dans 17 cas, par leur entourage dans 64 cas, et en pharmacie dans 12 cas. Elles ont été effectuées le matin au lever et le soir au coucher, contrôlées à deux reprises à chaque fois selon les recommandations internationales. Nous avons pris soin d'apprendre aux patients et à leur entourage la technique d'utilisation des appareils de mesure à travers de multiples démonstrations. Les appareils de mesures employés étaient de type électronique, à brassard huméral et disposant d'un système de mise en mémoire des valeurs enregistrées. Les valeurs des pressions artérielles ont été consignées dans des carnets de surveillance individuels précisant la date et l'heure exacte de la mesure. Nous avons distingué trois types d'HTA: L'HTA prédialytique, mesurée avant le branchement des patients; - l'HTA intradialytique, qui est une HTA prédialytique maintenue pendant la séance de dialyse ou une HTA qui survient de novo; -pendant la séance de dialyse chez un patient normotendu avant le branchement; -l'HTA post-dialytique immédiate.

Quant à l'HTA ambulatoire est celle relevée par les patients hors centre. Cependant, dans un souci d'objectivité scientifique, seules les mesures prises par le personnel soignant en centre ont été utilisées pour poser le diagnostic d'HTA. Nous avons défini l'HTA par des valeurs supérieures ou égales à 140mmHg pour la Pression Artérielle Systolique (PAS) et/ou 90mmHg pour la Pression Artérielle Diastolique (PAD) présentes sur au moins deux mesures prises et recontrôlées par le personnel soignant. Nous avons ainsi réparti nos patients en deux groupes: - groupe 1: patients hypertendus (groupe « HTA+ »), présentant une HTA prédialytique ou intradialytique, avec ou non persistance d'une HTA ambulatoire permanente; - groupe 2: patients non hypertendus (groupe « HTA - »). Dans le groupe « HTA+ », chez chaque patient, nous avons calculé la moyenne des tensions artérielles mesurées.

Puis, en comparant le groupe « HTA+ » au groupe « HTA - », nous avons pu mettre en exergue les FDR de l'HTA. Les comparaisons des deux groupes de patients ont porté sur les paramètres: -Anthropométriques (âge, sexe, niveau d’éducation); - Cliniques (néphropathie initiale, tares associées, symptomatologie clinique, indice de masse corporelle IMC, prise médicamenteuse, respect des règles hygiéno-diététiques, niveau d'activité physique); - Biologiques (numération sanguine, désordres minéralo-osseux, électrolytiques, anomalies cardiovasculaires, bilan thyroïdien); -Dialytiques. (durée en dialyse, rythme et durée des séances, poids sec, prise de poids interdialytique) *NB: la PPID a été évaluée comme la différence de poids entre la fin d'une séance de dialyse et le début de la séance suivante*. L'analyse statistique a été réalisée à l'aide du logiciel SPSS 11.5: - Les variables quantitatives sont exprimées en moyennes-écarts-types quand elles répondent à la loi normale et en médianes-extrêmes quand elles sont hors la loi normale. Leur comparaison est réalisée à l'aide du test statistique de Student; - Les variables qualitatives sont exprimées en pourcentages. Leur comparaison est réalisée à l'aide du test de Chi deux; - La différence est jugée statistiquement significative pour un p< 0,05.

Scribner [1] a établi très tôt une liste de recommandations concernant le traitement non pharmacologique de l'HTA en HD [1]. Celle-ci repose sur: - la diminution progressive du poids sec (PS) par une ultrafiltration adaptée; - le respect de la balance sodée durant la séance de dialyse; - la réalisation de séances d'HD de durée suffisante; - le suivi d'une diététique hyposodée; - et enfin l'arrêt progressif des médicaments anti-hypertenseurs lorsque toutes ces mesures sont bien installées et la correction de l'HTA amorcée. Aussi, lors de la deuxième phase, qui a duré deux mois, les patients du groupe 1 (groupe « HTA+ ») ont bénéficié d'une prise en charge basée sur les recommandations de Scribner [1] ainsi que sur les résultats de notre enquête statistique.

Afin d'inculquer à nos patients le respect du régime hyposodé, nous avons mis en place une stratégie d’éducation thérapeutique (ETP) comprenant plusieurs étapes. Aussi, nous avons tout d'abord multiplié les consultations individuelles afin de pouvoir établir un diagnostic pédagogique pour chaque patient, à savoir préciser son niveau de connaissance et de conscience vis-à-vis de sa maladie, son degré d'observance thérapeutique et des règles hygiéno-diététiques, les éventuels obstacles à braver. Nous avons ensuite élaboré un programme d’éducation thérapeutique basé sur le « modèle constructiviste », qui correspond à un travail de groupe par le biais d'expressions et de modules d'activités, et pour lequel nous avons opté pour l'esprit d’équipe et la notion d'entraide qu'il développe. Nous l'avons associé à une pédagogie allostérique c'est-à-dire qui tient compte des acquis des patients, compose avec leurs croyances en essayant de leur inculquer le savoir « avec » mais aussi « contre » leurs conceptions [2]. Concrètement, nous avons organisé des séances d'apprentissage bi-hebdomadaires d'une durée de deux heures, par petits groupes de 4 à 5 patients « voisins » pendant les séances de dialyse, lors desquelles nous abordions, en dialectal marocain, de façon illustrée et la plus ludique possible, différents sujets: l'IRCT en dialyse comme entité à part, avec ses spécificités, ses contraintes au quotidien, ses complications, son retentissement psychoaffectif, l'activité physique et la prise médicamenteuse en dialyse, mais surtout, l'observance du régime hyposodé, défini comme une consommation inférieure ou égale à 6g de NaCl par jour. Chaque médecin, aidé d'un infirmier, disposait de différents outils: -la mise en situation fictive d'un repas familial, d'une réunion, d'une fête religieuse, d'un repas de rupture de jeûne, l'objectif étant de faire changer les habitudes alimentaires des patients lors de ces repas de coutume copieux et bien assaisonnés; - des jeux de devinettes et de tâtonnement portant sur les teneurs en sel des aliments les plus consommés dans les foyers marocains, avec vérification grâce aux étiquettes des aliments (beurres, beurres rances, fromages, eaux minérales); - des jeux de recherche de « l'intrus » sur des photos de tables de repas; - l'estimation individuelle ou collective de la quantité de sel requise dans une recette classique de la cuisine marocaine, avec propositions d'alternatives culinaires; - et l’évaluation du niveau de compréhension des patients par l’élaboration de jeux mettant en compétition des équipes de patients aléatoirement formées.

Nous tenions néanmoins compte des individualités de chacun en termes de besoins, de sensibilité, de susceptibilité et de niveau de compréhension dans le but d'optimiser l'acquisition du savoir, et cela surtout compte tenu du nombre important de patients analphabètes dans notre population. Par ailleurs, un livret d'ETP a été créé pour chaque patient permettant d'assurer la traçabilité de l'ETP, d’évaluer les acquis, les actions restant à mener et l’évolution des différentes dimensions biologique, clinique, sociale et psychologique. Le deuxième effort réalisé fut le passage à trois séances de dialyse par semaine. Pour des raisons logistiques, ceci n'a concerné que 13 de nos patients du groupe « HTA+ », ce qui a élevé le nombre de patients dialysés 3 fois par semaine à 26, contre 31 dialysés 2 fois par semaine. Le choix se porta sur les plus hypertendus d'entre eux, c'est-à-dire ceux dont la PAS était supérieure ou égale à 180 mmHg.

Enfin, nous avons entrepris une réduction du PS chez les 48 patients dont l'HTA persistait en fin de séance et cela à raison de ½Kg /15 jours dans les limites de la tolérance hémodynamique. En effet, nous avons arrêté notre baisse du PS dès l'apparition de symptômes pendant les séances tels que l'hypotension artérielle, les malaises, nausées, crampes douleurs, ou encore vomissements. A noter que tous nos patients sont dialysés avec des bains sans acétate conformes aux normes internationales et que les conductivités des dialysats ont toujours été adaptées aux natrémies des patients. Durant la troisième phase, nous avons évalué les retombées de ces mesures non pharmacologiques en termes de prise de poids interdialytique (PPID) et de chiffres tensionnels. La mesure et la collecte des pressions artérielles a été effectuée avec la même méthodologie que pour la première phase. Quant aux mesures de poids, elles ont toujours été effectuées sous la supervision d'un membre du personnel soignant, avec le même pèse-personne qu'en phase 1, standardisé et taré.

## Résultats

Nous n'avons établi aucun critère d'exclusion ni essuyé aucun refus de participation des patients, si bien que l'ensemble de nos hémodialysés chroniques ont été inclus dans notre travail. Notre population totale comptait donc 93 hémodialysés chroniques, dont l’âge moyen était de 50,8±15,5 ans. Les sujets âgés (d’âge supérieur ou égal à 65 ans) représentaient 20,4% de l'ensemble de nos patients (n = 19). Le sexe ratio H/F était de 0,978 (46H/47F) et l'ancienneté moyenne en hémodialyse de 88±54,03 mois. 58 de nos patients avaient un rythme de 2 séances de 5 heures de dialyse par semaine, tandis que les 35 restants étaient dialysés à raison de 3 fois par semaine pendant une durée de 4 heures par séance. Les étiologies de l'insuffisance rénale chronique terminale (IRCT) de ces patients sont résumées dans le Tableau 1. Le taux moyen d'hémoglobinémie de notre population était de 10,6±3,1 (6-16,5) g/dl. Parmi nos 93 patients: -52, soit 55,9%, étaient anémiques (Hb

**Tableau 1:** Néphropathies initiales dans notre population d'hémodialysés chroniques

Néphropathie causale	n = 93	*%*
Indéterminée	44	*47,31*
PKR	15	*16,12*
Diabétique	12	*12,9*
Vasculaire	9	*9,67*
NTIC	8	*8,6*
NGC	5	*5,37*

PKR: Polykystose rénale; NTIC: Néphrite tubulo-interstitielle chronique; NGC: Néphropathie glomérulaire chronique

Les résultats de la phase I ont été les suivants: -Une TA supérieure aux seuils fixés a été retrouvée lors de 231 séances de dialyse sur 442 (Tableau 2), ce qui correspond à 57 patients soit une moyenne de 4,05±0,95 [3–6] séances avec HTA + /patient en deux semaines. Seuls 9 de ces patients (15,7%), dont 7 dialysés 2 fois/semaine et 2 dialysés 3 fois/semaine, ressortaient régulièrement de la séance de dialyse avec une TA correcte. Tous les autres patients restaient hypertendus au débranchement. Ainsi, l'HTA découverte en centre était: -Pré-dialytique lors de 231 séances (chez les 57 patients); - Intra-dialytique à 219 séances (chez 55 patients); - et post-dialytique durant 190 séances (chez 48 patients). Ces résultats sont consignés dans le Tableau 3. Cette HTA était d'ordre systolique, systolo-diastolique ou encore diastolique seul dans respectivement 80,34%, 12,6% et 6,98% des cas. La PAS moyenne était ainsi de 172,75±17,69 (145-220) mmHg. Quant aux 1720 mesures de PA effectuées hors-centre, parmi les 1088 faites par les 57 patients hypertendus, seules 74 étaient normales (soit 6,8%): elles concernaient les 9 patients normotendus en fin de séance et sur au moins une mesure le jour suivant la dialyse. Ainsi, la prévalence de l'HTA dans notre population d'hémodialysés chroniques était de l'ordre de 61,3%.

**Tableau 2:** Profil des séances de dialyse dans le groupe 1 «HTA + » (n = 57)

	Rythme de 2 séances/semaine	Rythme de 3 séances/ semaine
*Nombre de patients hypertendus*	*44*	*13*
Nombre de séances surveillées/semaine	88	39
Nombre de séances surveillées/2 semaines	176	78
*Total des séances surveillées*	*254*	
Nombre de patients ayant présenté 3 épisodes d'HTA en centre en 2 semaines	16	-
Nombre de patients ayant présenté 4 épisodes d'HTA en centre en 2 semaines	28	2
Nombre de patients ayant présenté 5 épisodes d'HTA en centre en 2 semaines	-	3
Nombre de patients ayant présenté 6 épisodes d'HTA en centre en 2 semaines	-	8
*Nombre de séances où une HTA a été notée en 2 semaines*	*160*	*71*
*Total des séances pendant lesquelles une HTA a été notée*	*231*	

**Tableau 3:** Type d'HTA notée lors des séances dans le groupe 1 «HTA + » (n = 57)

		HTA pré-dialytique		HTA intra-dialytique		HTA post-dialytique	
Nombre de séances	*Nombre de patients dialysés 2 fois/semaine*	160	*44*	154	*44*	132	*37*
Nombre de séances	*Nombre de patients dialysés 3 fois/semaine*	71	*13*	65	*11*	58	*11*
Total des séances	*Total des patients*	231	*57*	219	*55*	190	*48*

Les caractéristiques des 57 patients diagnostiqués hypertendus étaient les suivantes: -un âge moyen de 60,8±13,76 (31-83) ans (19 patients, soit 33,3%, étaient âgés de plus de 65 ans); - un sexe ratio de 1,19 (soit 31H/26F); - un rythme de 2 et 3 séances d'hémodialyse par semaine chez respectivement 44 et 13 d'entre eux; - une PPID moyenne de 3,52±0,51 (2,1-4,98) Kgs; -70,1% d'entre eux (n = 40) disaient respecter scrupuleusement les règles hygiéno-diététiques (RHD) recommandées; - De plus, 32 patients (56,1%) suivaient un traitement anti-hypertenseur, constitué d'une, deux ou trois classes pharmacologiques dans respectivement 59,3% (n = 19), 34,4% (n = 11) et 6,2% (n = 2) des cas; - 30 patients étaient anémiques, avec une hémoglobinémie moyenne à 9,9 ± 3 (6-15,6) g/dl; - 12 (21%) étaient sous érythropoïétine recombinante, 12 sous fer injectable, 16 sous fer per os et enfin 7 sous supplémentation en vitamine B12; - enfin, la répartition de leurs néphropathies causales d'IRCT est représentée dans le Tableau 4. En comparant le groupe 1 « HTA+ » au groupe de patients « contrôle », l'analyse statistique nous a permis de retenir comme FDR l’âge (p<0,0001), la PPID importante (p<0,0001), le non respect des règles hygiéno-diététiques (p=0,014) et le rythme de deux séances de dialyse par semaine (p<0,0001) (Tableau 5).

**Tableau 4:** Répartition des néphropathies initiales dans le groupe 1 « HTA + » (n = 57)

Néphropathie causale	n = 57	*%*
Indéterminée	30	*52,6*
PKR	8	*14,03*
Diabétique	6	*10,52*
Vasculaire	6	*10,52*
NTIC	4	*7,01*
NGC	3	*5,26*

**Tableau 5:** Analyse statistique des FDR d'HTA dans notre population d'hémodialysés chroniques

Paramètres	HTA + : n = 57	HTA - : n = 36	*P*
**Age (ans)**	60,8±13,76	39,11±13,37	**<0,001**
**Sexe (n)**	31H/26F	15H/21F	*NS*
**Néphropathie initiale (n)**			
*Indéterminée*	30	14	*NS*
*Diabétique*	6	6	*NS*
*Vasculaire*	6	3	*NS*
*PKR*	3	5	*NS*
*glomérulaire*	8	7	*NS*
*NTIC*	4	1	*NS*
**Ancienneté en HD (mois)**	96,22±53,31	77,11±53,82	*NS*
**PPID (Kgs)**	3,52±0,51	2,68±0,68	**<0,001**
**Respect des RHD : n** ***(%)***	40 *(70,1)*	33 *(91,6)*	**0,014**
**2 séances de dialyse/semaine: n** ***(%)***	44 *(77,2)*	12 *(33,3)*	**<0,001**

En phase 2, nous avons entrepris l'application des recommandations de Scribner pour tenter une réduction non pharmacologique de l'HTA chez les 57 patients hypertendus. Le premier effort fut basé sur l'explication, l'installation et l'observance des règles hygiéno-diététiques imposées par la dialyse chronique. Ainsi, aux termes d'un diagnostic pédagogique rigoureux, il a été démontré que: - 87,7% (n = 50) des patients étaient d'un niveau socio-économique bas; - 80,7% (n = 46) avaient un niveau d'instruction insuffisant; - tous étaient entourés d'au moins un adulte proche instruit et impliqué dans l'organisation de leur hygiène de vie quotidienne. Puis, notre programme d'ETP comme expliqué précédemment s'est étalé sur deux mois à raison de deux séances d'apprentissage de deux heures par semaine pour chaque patient, soit un nombre total de 32 heures d'apprentissage par patient. Au final, nous avons atteint 92,9% de respect des RHD selon les dires des patients, ce que confirme également la baisse de la PPID (n = 53). Parmi les 53 patients respectant ces règles, 17 avaient plus de 65 ans.

Le deuxième effort réalisé fut le passage à trois séances de dialyse par semaine, qui a été possible chez 26 patients, contre 13 au départ. La réduction du PS a concerné 48 patients qui présentaient une HTA persistante en post-dialyse, avec au final une baisse moyenne de 0,98 ± 0,42 Kg. Ainsi, le PS est resté inchangé chez 9 patients, il a baissé d'une valeur de 0,5kg chez 15 patients, 1Kg chez 21 patients, 1,5 Kgs chez 10 patients et 2 Kgs chez 2 patients. Pendant la troisième phase, nous avons évalué les résultats de notre prise en charge. Il s'est avéré que 8 patients, 5 femmes et 3 hommes parmi lesquels 5 d’âge supérieur ou égal à 65 ans, sont passés dans la catégorie « normotendus ». En effet, leurs chiffres tensionnels se sont normalisés, à toutes les mesures pour 6 d'entre eux et au-dessus de la normale sur une seule mesure pour les 2 restants. La prévalence de l'HTA dans notre population est ainsi passée à 52,7% (n = 49). Dans le groupe « HTA+ » désormais constitué de 49 patients, parmi lesquels 14 âgés de plus de 65 ans, des changements dans le profil tensionnel se sont également produits: - la TA moyenne a baissé d'une valeur supérieure ou égale à 20 mmHg chez 24,5% des patients (n = 14), dont 5 hommes et 9 femmes (3 patients âgés); - de moins de 20 mmHg chez 26,3% des patients (n = 15), dont 6 hommes et 9 femmes (6 patients âgés); - et est demeurée inchangée dans 49,2% des cas (n = 20), dont 17 hommes et 3 femmes (5 patients âgés).

La moyenne de la PAS est ainsi passée à 166,12±17,05 (140-200) mmHg. Parmi les 8 patients passés dans le groupe « sans HTA», 2 étaient des « hypertendus permanents », c'est-à-dire aussi bien en ambulatoire que pendant les séances de dialyse. En ce qui concerne la PPID, les efforts de restriction hydrosodée ont permis sa diminution dans 80,7% des cas (n = 46 patients) répartie comme suit: - une baisse inférieure ou égale à 0,5Kg chez 13 patients; - entre 0,5 et 1 kg chez 17 patients; - entre 1 et 1,5 kgs chez 14 patients; - et strictement supérieure à 1,5 kgs chez deux patients.

Ceci revient à une baisse moyenne de 0,98±0,42 Kgs. A l'inverse, il n'y a pas eu de baisse de la PPID chez 11 patients (19,2%). Parmi les 8 patients devenus « normotendus », 3 étaient traités par une seule classe d'antihypertenseur et ont pu en être sevrés sans survenue d'effet rebond de leurs PA. Parmi les 49 patients restés hypertendus en phase 3, 29 (50,8%) étaient sous traitement antihypertenseur et le sont restés: - 16 (28%) sous une seule classe thérapeutique; - 11 (19,2%) sous deux classes; - et 2 (3,5%) sous 3 classes. Nous constatons par ailleurs révélé que l’âge avancé a été un facteur de bonne adhésion et de meilleure réponse à notre programme d'ETP, même si cette observation n'a pas pu être vérifiée statistiquement.

## Discussion

Notre étude a objectivé une prévalence de l'HTA dans notre population d'hémodialysés chroniques de 62,3%. Notre objectif ayant consisté en la réduction de ce pourcentage, nous avons établi une stratégie basée sur les recommandations de Scribner en trois volets: -la réduction du PS; - l'augmentation de la quantité de dialyse; - et le respect du régime hyposodé afin d'agir sur les variations volumiques en interdialyse et donc sur la PPID; -Pour ce dernier volet, un programme d'ETP bien codifié nous a été d'une grande utilité. Au final, nous avons pu réduire la prévalence de l'HTA dans notre population à 52,7%.

Les enquêtes épidémiologiques réalisées au cours de cette dernière décennie concluent toutes à une prévalence élevée de l'HTA en HD, comprise entre 55 et 85% selon les études [3]. Cependant, le plus inquiétant est que cette prévalence a augmenté au cours des dernières années. En effet, dans les années 1970, il était possible de passer de 90% à moins de 30% d'hypertendus après uniquement six mois d'HD bien conduite [4] et pour cause, les patients insuffisants rénaux qui arrivaient au stade de dialyse avaient un âge moyen d'environs 50 ans [5]. Par contre, depuis les années 1990, il s'est avéré que le contrôle tensionnel par la dialyse est devenu insuffisant, et on peut invoquer deux raisons principales à cela: 1) tout d'abord, il y a une fois de plus la question de l’âge, puisque les patients qui arrivent au stade de dialyse sont de plus en plus âgés [6]. Dans notre série, les sujets âgés représentaient le cinquième de notre population hémodialysée et le tiers de notre groupe 1 « HTA+ ». Ainsi, tous nos sujets âgés étaient hypertendus. 2) La deuxième explication est que les néphropathies vasculaires constituent actuellement entre 35 et 40% des néphropathies causales d'IRCT, tandis qu'elles ne représentaient que 5% de celles-ci dans les années 1970 [6]. Or, ces néphropathies vasculaires ont pour facteurs de risque reconnus le diabète et l'HTA indépendamment de l'HTA secondaire à l'insuffisance rénale. Ainsi, les patients concernés par cette situation ont un passé de diabète avec des lésions vasculaires endothéliales et/ou d'HTA antérieur à l'insuffisance rénale. Par conséquent, ces patients ne corrigent pas leurs chiffres tensionnels même après l'entrée en dialyse, requérant le plus souvent un traitement antihypertenseur. Dans notre travail, les néphropathies vasculaires et diabétiques représentaient 21% des néphropathies causales.

Toutefois, les données épidémiologiques rapportées dans la littérature sont à relativiser puisque les différents auteurs ne se basent pas sur une même définition de l'HTA. En effet, certains se réfèrent à la pression artérielle moyenne (PAM), d'autres à la pression artérielle systolique pré-dialytique, en fixant la valeur-seuil tantôt à 140 mmHg, tantôt à 150 mmHg. De même que la définition de l'HTA dépend de l’âge et des comorbidités de la population étudiée. Ainsi, en l'absence de complications cardio-vasculaires, la normotension correspond à une PAS prédialytique au repos inférieure à 130/80 mmHg selon les recommandations du JNC7. En revanche, la cible recommandée pour une personne âgée porteuse d'athérosclérose est plutôt de 140-150 mmHg [7]. Par ailleurs, les patients anciennement hypertendus mais désormais contrôlés sous traitement anti-hypertenseur, peuvent être classés dans la catégorie « normotendus » ou dans celle des « hypertendus » selon les auteurs [8].

Concernant la méthode de mesure de la PA, il n'existe actuellement aucune étude de cohorte de patients dialysés qui ait pu déterminer la prévalence de l'HTA en fonction de la MAPA [9]. Dans notre travail, la réalisation d'une MAPA chez tous nos patients n’étant pas aisée pour des raisons logistiques, nous avons opté pour une mesure horaire de la PA durant les séances de dialyse. Nous avons complété l'appréciation du profil tensionnel de nos patients par des mesures biquotidiennes effectuées par ceux-ci les jours de non dialyse.

Nous avons distingué plusieurs types d'HTA: - l'HTA prédialytique; - l'HTA intra-dialytique; - l'HTA post-dialytique immédiate; - et l'HTA ambulatoire permanente. Cependant, nous avons basé le diagnostic d'HTA sur les mesures de PA effectuées en centre. Puisque nos patients hypertendus en centre sont en fait en majorité porteurs d'une HTA permanente, nous pouvons affirmer que les chiffres de PA que nous avons enregistrés chez nous ne sont pas la conséquence d'un « effet blouse blanche ». L'HTA en IRC mais aussi en HD se caractérise par une élévation de la PAS tandis que la PAD reste le plus souvent normale voire basse. Ceci augmente alors la différentielle ou pression pulsée, ce qui constitue un facteur de risque cardio-vasculaire indépendant [10–14]. Dans notre série, l'HTA était systolique isolée dans plus de 80% des cas.

Cause ou conséquence des néphropathies, l'HTA en dialyse reste fortement liée au volume hydrosodé, le sel étant considéré aujourd'hui chez l'insuffisant rénal comme une véritable « toxine urémique » [15]. Aussi, d'un point de vue physiopathogénique, l'inflation du volume extracellulaire (VEC) et l'augmentation des résistances vasculaires périphériques (RVP) qui en découlent sont les mécanismes principaux de l'HTA aux différents stades de l'IRC [16, 17]. Comme cela a été déjà évoqué expérimentalement, le sodium interfère avec la réactivité endothéliale sous la dépendance de l'oxyde nitrique et de son inhibiteur l'ADMA (asymetrical dimethyl arginine) [18, 19]. Les résistances périphériques chez les patients hémodialysés hypertendus ont été étudiées par Kim et al [20] et retrouvées fréquemment augmentées. De la même façon, Katzartski et al [21] ont montré que les patients hémodialysés hypertendus ont des résistances périphériques plus élevés que les patients normotendus en dialyse longue.

Toutefois, Scribner [1] a été l'un des premiers cliniciens à se baser sur l'implication du VEC et des RVP dans l'HTA de l'hémodialysé et de ce fait, à poser les deux pierres angulaires du traitement non pharmacologique de l'HTA. Il a ainsi préconisé la correction du VEC par ultrafiltration et une diététique hyposodée. Il tenta l'expérience sur le premier patient Clyde Schields, qui survécut 11 ans à son IRCT. Le terme de « poids sec » (PS) ne verra le jour que plus tard, grâce à Thomson et al. [22] en 1967; et c'est ainsi qu'on parle désormais de « méthode du PS ». Les mesures associées nécessaires sont le respect de la balance sodée au cours de la séance, la durée suffisante de la séance de dialyse et l'arrêt progressif des antihypertenseurs lorsque ces mesures sont en place. Par contre, pour ce qui est des RHD, l'habitude très répandue de conseiller une restriction des apports hydriques est inefficace et illusoire si le conseil de restriction sodée n'y est pas associé [23].

Cela dit, notre travail, au même titre que d'autres de la littérature, constate que la correction du VEC n'aboutit pas automatiquement à la normalisation de la TA et qu'il existe un intervalle entre les deux appelé « lag phenomenon », qui crée durant environ six mois une situation souvent délicate à gérer [24]. Le mécanisme physiopathologique exact du « lag phenomenon » n'est pas encore élucidé, mais on retient néanmoins deux hypothèses de poids: - la première est que le PS varie en fonction des évènements cataboliques et anaboliques intercurrents; - la deuxième est que la correction du VEC et de ses perturbations hémodynamiques n'engendre qu'une correction très progressive du remodelage vasculaire et donc des RVP [25, 26]. Ainsi, au bout de trois mois, nous n'avons pu baisser la prévalence de l'HTA dans notre série que de 10%, certainement à cause de l'impact péjoratif de la fréquence hebdomadaire de la dialyse, mais probablement aussi en raison de ce phénomène de décalage décrit dans la littérature.

### Apport de l’éducation thérapeutique (ETP) appliquée à la méthode du PS dans notre série

De nos jours, la méthode du PS en hémodialyse ne peut plus se concevoir sans l'instauration d'une réelle stratégie d’éducation thérapeutique des patients. En effet, on peut considérer comme indéniable sur le plan de l'efficacité thérapeutique, la valeur de l'acquisition de connaissances par les patients quant aux subtilités de leur maladie, particulièrement quand celle-ci est chronique [27]. Ceci est d'autant plus vrai pour les patients de dialyse, chez qui les contraintes imposées par l'IRCT ne doivent avoir d’égale que l'intensité de leur implication dans leur processus de survie et la justesse de leur prise en charge tant physique que psychologique. Chez nos patients, en dépit de tout l'arsenal thérapeutique dont nous pouvions disposer ainsi que de notre capacité à jongler avec leurs paramètres dialytiques, l'atteinte de nos objectifs tensionnels ne pouvait passer que par un effort et une implication consentis de ces derniers, qui vont de paire avec une bonne connaissance et conscience de leur part des différents aspects de leur maladie.

Il fut un temps où le paradigme de l'ETP était le biomédical, c'est-à-dire qu'on estimait que la maladie avait un substrat organique que le médecin se devait d’éradiquer en prodiguant des soins: le « bon patient » était alors considéré comme le « bon observant » [2]. Bon nombre d'idées ont été acquises depuis, et l'on sait actuellement que l'ETP constitue une nouvelle appréciation de la relation « médecin-malade ». Ainsi, «le but de l’éducation thérapeutique est que les patients (et leurs familles) comprennent leur maladie et leur traitement, collaborent avec l’équipe soignante et prennent la responsabilité de leur traitement comme un moyen de maintenir et d'améliorer leur qualité de vie [28] ».

En France, l'ETP est inscrite dans la Loi « Hôpital, Patients, Santé, Territoires » HPST, dite Loi Bachelot et promulguée en 2009 [29]. Le système de santé marocain a pour l'instant d'autres priorités. Néanmoins, nous avons décidé de faire de l'ETP notre cheval de bataille dans la réduction du taux d'HTA dans notre série, convaincus de son impact sur le succès de notre prise en charge à l'instar d'autres études [30–32]. Une ETP bien conduite comprend plusieurs étapes. Tout d'abord un diagnostic pédagogique en parallèle au diagnostic clinique qui consiste à repérer les besoins du patient, les compétences à acquérir ou à mobiliser, les obstacles potentiels [2]. Pour notre part, nous avons eu recours à des séances prolongées de dialogue avec nos patients dans le but d’établir un climat de confiance et d'atteindre les objectifs du diagnostic pédagogique. Puis, il faut établir en collaboration avec le patient un programme personnalisé d'ETP envisageant le type de situations à installer [2]. Ensuite, il faut mettre en place les séances d'ETP individuelles et/ou collectives et enfin, évaluer le patient c'est-à-dire faire le point sur ce qu'il a compris, appris, sait faire et comment il vit sa maladie au quotidien [2]. Dans notre travail, nous avons organisé des séances de discussion constitués de 4 à 5 patients voisins pendant les séances de dialyse. Durant celles-ci, nous traitions de l'IRCT en dialyse comme entité à part, avec ses spécificités, ses contraintes au quotidien et ses complications, mais surtout des principes de l'hygiène de vie d'un patient de dialyse en termes de régime, d'activité physique et de prise médicamenteuse.

La tenue de cahiers d'ETP permet un suivi de l'avancement du processus d'apprentissage ainsi que la transmission de consignes entre membres du personnel soignant. Afin d'unifier la gestion de nos patients, nous avons intégré nos cahiers d'ETP aux cahiers standards de surveillance d'HD chronique. A noter que nous n'avons rencontré aucune réticence à ce programme de la part de nos patients ou de leur entourage. Par ailleurs, nous constatons globalement une meilleure adhésion au programme d'ETP dans le rang des femmes, comme semble d'ailleurs le confirmer l’évolution en phase 3 des chiffres tensionnels dans le nouveau groupe « HTA+ » composé de 49 patients.

On constate enfin que parmi les 19 patients âgés de plus de 65 ans et hypertendus au départ, 5 sont devenus normotendus en phase 3 et 9 autres restés hypertendus ont tout de même baissé leurs chiffres tensionnels. L’âge avancé, qui sous-entend une prise en charge plus étroite des patients par leur famille étant donné leur relative perte d'autonomie, a donc été un facteur de bonne adhésion et de meilleure réponse à notre programme d'ETP, et cela paradoxalement malgré les altérations hémodynamiques, cardiovasculaires et les comorbidités qui accompagnent le vieillissement. Au final, le principal problème que nous avons rencontré au cours de notre travail dans l'application de ces mesures n'a été ni le manque de motivation ou d'adhésion des patients à notre programme d'ETP, ni la non régularité de ceux-ci dans la prise des mesures de PA hors centre, mais bien un problème de moyens logistiques et financiers rendant impossible la généralisation du rythme de trois séances de dialyse à un plus grand nombre. Nous avons en effet dû choisir parmi les patients les plus hypertendus d'entre eux pour bénéficier de cette mesure. Nous poursuivons d'ailleurs actuellement notre travail d'ETP, ainsi que l'application de la méthode du poids sec, mais sans pouvoir augmenter davantage pour le moment le nombre de patients dialysés trois fois par semaine, puisque notre centre est arrivé à saturation.

## Conclusion

La « méthode du PS » peut permettre de corriger l'HTA en HD, mais ce succès ne peut se concevoir sans un effort pédagogique soutenu de la part de l'ensemble de l’équipe soignante, d'où l'intérêt de planifier une ETP. Cependant, cette méthode a tendance à être plus ou moins délaissée au profit d'un traitement pharmacologique qui plus est peu efficace. C'est la raison pour laquelle Scribner remet en 2002 un écrit qui résonne comme un signal d'alerte [33], dans lequel il se désole de l’épidémiologie actuelle de l'HTA en HD malgré toutes les avancées techniques et thérapeutiques. Il incrimine l'engouement de la plupart des praticiens pour les thérapies médicamenteuses. Aussi, il appelle la communauté scientifique à se fixer pour défi la réhabilitation de la méthode non pharmacologique principalement auprès des jeunes et futurs néphrologues.
